# Acorn Bread: The Synergy of Acorn Flour, Sustainability, and EU Organic Standards

**DOI:** 10.3390/foods15101625

**Published:** 2026-05-07

**Authors:** Petra Lončarić, Marko Jukić, Darko Velić, Jasmina Lukinac

**Affiliations:** Faculty of Food Technology Osijek, Josip Juraj Strossmayer University of Osijek, F. Kuhaca 18, 31000 Osijek, Croatia; ploncaric@ptfos.hr (P.L.); marko.jukic@ptfos.hr (M.J.); dvelic@ptfos.hr (D.V.)

**Keywords:** *Quercus* spp., bioactive compounds, circular economy, EU legislation, food security, functional breadmaking, gluten-free, organic production, rheological properties, underutilized resources

## Abstract

Acorns (*Quercus* spp.) have a long history of use in human nutrition and represent an underutilized plant resource with considerable potential for sustainable food systems. In recent years, acorn flour (AF) has attracted scientific interest as an innovative ingredient, particularly in breadmaking, due to its lack of gluten, favorable nutritional profile, and technological functionality. This review summarizes bread made with AF as a sustainable and innovative food product, with particular emphasis on its technological properties, including rheological behavior, sensory attributes, and nutritional profile. To our knowledge, this review is among the first to integrate an analysis of recent technological advancements in breadmaking with the current European Union regulatory framework, specifically EU Organic Regulation 2018/848. By bridging the gap between food technology and legislative requirements, the review outlines a comprehensive pathway for the commercialization of acorns as a certified organic resource. It concludes that although acorns have significant potential to enhance food security and sustainability, further standardization and increased consumer awareness are necessary to ensure their successful market integration.

## 1. Introduction

Species of the genus *Quercus* (family *Fagaceae*) are widely distributed across Europe, North Africa, Asia, and North America and represent one of the most ecologically significant forest taxa in temperate and Mediterranean ecosystems [1,2,3,4,5]. Their fruit, the acorn, is a single-seeded nut whose chemical composition varies considerably among species due to phylogenetic, environmental, and climatic factors. Historically, acorns played a substantial role in human nutrition, particularly in regions where oak forests were abundant and cereal cultivation was limited [6].

Archaeobotanical evidence and historical records confirm the widespread use of acorn flour (AF), particularly for breadmaking [7,8,9]. However, with the intensification of cereal agriculture, acorns gradually became associated mainly with animal feed and subsistence economies [10,11]. In recent years, this perception has begun to change.

Europe relies heavily on wheat, producing over 130 million tons a year. However, recent droughts and heatwaves have shown that depending on a single crop is risky. To build a more stable food system, we need to diversify our ingredients. Acorns represent a promising alternative resource: they grow easily in dry conditions and align with the EU’s goals for sustainable farming. Furthermore, forest-derived foods, including products obtained from trees (such as acorns), are accessible and highly nutritious, thereby contributing to enhanced food security. This particularly applies to the least developed regions [9,12]. AF represents a nutritionally valuable product, as its dietary fiber (DF), lipid and mineral contents exceed those in wheat [13,14,15,16,17,18,19,20]. Beyond nutritional benefits, acorns also offer economic potential. According to Inácio et al. (2024), their relevance extends beyond consumer-related factors such as affordability and accessibility to include significant industrial applications, where they may serve as a sustainable raw material contributing to a greener and more resilient economy [21]. Although acorn yields are subject to interannual variability due to masting behavior, diversified sourcing across species and regions may help mitigate supply fluctuations [22].

Beyond their historical significance, acorns have a distinctive nutritional and phytochemical profile. They contain significant amounts of starch, DF, lipids rich in unsaturated fatty acids (UFAs), minerals, and phenolic compounds with antioxidant activity [23,24,25,26]. Several studies have linked acorn-derived phenolics to antioxidant, anti-inflammatory, antimicrobial, and potential cardioprotective effects [27,28,29]. Nevertheless, these health-related claims require careful interpretation and further clinical validation.

A central technological challenge in acorn utilization is their tannin content. Tannins contribute to bitterness and may have protein-binding effects traditionally regarded as anti-nutritional. At the same time, they are potent bioactive antioxidants [30]. The key technological objective is not complete removal, but controlled modulation—reducing sensory drawbacks while preserving functional bioactivity.

Recent scientific research has focused on AF as a gluten-free (GF) ingredient for breadmaking [23,24,31,32,33,34,35]. Gluten-free bread (GFB) production remains technologically challenging due to the absence of the viscoelastic gluten network, which typically results in lower volume, poorer crumb structure, and inferior texture compared to wheat bread [36]. AF presents an intriguing alternative: it is naturally GF, is generally not considered a novel food under the current EU framework, and provides DF, lipids, and phenolic compounds that may enhance both the nutritional value and functional properties of GF formulations [31].

Alongside technological considerations, regulatory alignment has become increasingly important. Regulation (EU) 2018/848 on organic production defines organic farming as a system combining environmental stewardship, biodiversity preservation, resource efficiency, and high production standards [37]. Acorn harvesting from biodiverse oak forests—ecosystems typically characterized by minimal external inputs and long-term carbon sequestration—appears inherently compatible with these principles [38]. In addition, this approach can contribute to several Sustainable Development Goals (SDGs), particularly SDG 2 (Zero Hunger), by supporting diversified and resilient food sources; SDG 12 (Responsible Consumption and Production), through the use of underutilized forest resources and promotion of sustainable supply chains; SDG 13 (Climate Action), via carbon sequestration in forest ecosystems; and SDG 15 (Life on Land), by encouraging the sustainable management of forest biodiversity [39]. Furthermore, developing short supply chains and regional valorization of forest resources supports circular economy objectives and rural resilience.

Despite the growing body of technological research on acorn-based bakery products, the literature remains fragmented. Most studies address isolated aspects—rheological behavior [24,31,35,40,41,42], nutritional composition [3,33,43], or sensory properties [31,32,40,44]—without integrating these findings within a broader sustainability and regulatory framework. In particular, the compatibility of acorn processing and commercialization pathways with Regulation (EU) 2018/848 has not been systematically examined.

This review aims to evaluate acorn flour bread as a sustainable and nutritionally valuable food product, while examining its technological feasibility and regulatory compatibility within the European context.

## 2. Materials and Methods

This systematic review was conducted in accordance with the Preferred Reporting Items for Systematic Reviews and Meta-Analyses (PRISMA) guidelines. The methodology was designed to integrate a multidisciplinary analysis of acorn flour (AF) technology, functional nutrition, and EU organic legislation.

### 2.1. Search Strategy and Information Sources

A comprehensive systematic search was performed across three primary electronic databases: PubMed, Web of Science, and Scopus. The search covered literature published from January 1990 to February 2026, with no lower date restriction applied in the database search interface, as the topic warranted inclusion of foundational studies. The search strategy used Boolean operators (AND, OR) and the following keywords: “acorn flour,” “breadmaking,” “Quercus spp.,” “gluten-free,” “functional bread,” “acorn flour bread,” “sustainability,” and “Regulation (EU) 2018/848.”

### 2.2. Inclusion and Exclusion Criteria

To ensure high-quality data synthesis, the following inclusion criteria were applied:

- Peer-reviewed research articles and reviews focusing on the nutritional and phytochemical characterization of *Quercus* species.
- Studies investigating the technological and rheological impact of AF substitution in wheat-based and gluten-free (GF) bread formulations.
- Research addressing environmental sustainability metrics (e.g., CO_2_ emissions) of oak agroforestry compared to industrial cereal systems.
- Legal analyses regarding EU organic production standards and the commercialization of wild-collected forest products.

Exclusion criteria included studies focusing exclusively on animal feed without implications for human nutrition, articles not available in English, and grey literature without rigorous peer review.

### 2.3. Selection Process and Data Extraction

The selection process followed a four-stage PRISMA approach: identification, screening, eligibility assessment, and inclusion. After removing duplicates, titles and abstracts were screened for relevance to the synergy between acorn technology and sustainability. A total of 154 references were selected for qualitative synthesis and are summarized in Table 1, Table 2 and Table 3 to provide a coherent overview of the field.

## 3. Potential of Acorns as Functional Food Ingredients

### 3.1. Ethnobotanical and Historical Perspective of Acorns

Species of the genus *Quercus* have long contributed to human subsistence, particularly in temperate and Mediterranean regions [1,2,3]. Archaeobotanical evidence confirms the processing of acorns into flour as early as prehistoric times, as indicated by rotary querns and grinding tools recovered from Mediterranean and Iberian sites [7,8].

In Europe, the practice of balanophagy (acorn consumption) was historically documented in the Iberian Peninsula and parts of Italy [4,9,26]. Although often associated with periods of scarcity, acorn-based foods persisted culturally and re-emerged during times of economic hardship, including the two World Wars and the Spanish Civil War [3,5].

Beyond breadmaking, acorns have been used as roasted flour substitutes for coffee, for oil extraction, and in medicinal preparations [4,12,45]. These diverse applications underscore their historical versatility and support renewed interest in their functional revalorization.

### 3.2. Sustainability and Food Security Relevance of Acorns

The increasing global demand for food, combined with climate change and resource depletion, has intensified the search for resilient and underutilized plant resources [46,47]. Acorns are a promising candidate in this context due to several structural advantages:

- They originate from long-lived perennial systems.
- Oak forests require minimal agricultural inputs.
- Harvesting does not require land conversion.

Forest ecosystems provide services such as biodiversity preservation and carbon sequestration. Historically undervalued and primarily used for animal feed [10,11], acorns are now increasingly recognized as potential contributors to sustainable and circular food systems. Their valorization aligns with current strategies promoting diversification of plant-based raw materials.

### 3.3. Nutritional Composition of Acorns

The nutritional composition of AF, predominantly reported on a dry weight basis in the cited studies, depends on acorn species and is characterized by a high carbohydrate content (up to 84%), moderate lipid levels (8–13%), significant DF (approximately 11–18%), and relatively low protein content (4–5%) (4–5%) [3,15]. Starch is the dominant carbohydrate fraction (31–49%), with amylose content varying considerably depending on species and environmental conditions [11,48]. Furthermore, according to Regulation (EC) 1924/2006, AF fits the category of foods high in fiber [49]. This variability significantly influences functional and rheological properties.

Acorn lipids are rich in UFAs, particularly oleic acid (approximately 60%) and linoleic acid (approximately 15%) [15,50,51]. The fatty acid profile resembles that of olive oil in its predominance of monounsaturated fatty acids (MUFAs), potentially contributing to favorable nutritional attributes. However, high unsaturation also increases susceptibility to oxidative deterioration [52,53].

DF levels in AF exceed those found in refined wheat flour and are comparable to certain whole-grain (WG) cereals such as wheat and rye [15]. Given the established association between DF intake and reduced risk of cardiovascular disease and metabolic disorders [54,55,56], this represents a significant nutritional advantage. Martins et al. (2022) [3] reported that DF in AF is predominantly composed of insoluble fractions (approximately 10.3 g/100 g DW), while soluble DF is present in lower amounts (around 1.1 g/100 g DW). These values may vary depending on species and processing conditions. This distribution is both technologically and nutritionally relevant. Insoluble DF primarily contributes to water absorption and structural reinforcement in gluten-free systems, while soluble DF affects viscosity, starch digestibility, and glycemic response. The ratio of soluble to insoluble DF is therefore a key factor in determining the functional properties and potential health benefits of acorn flour-based products [3,57]. Insoluble DF is also associated with beneficial physiological effects, particularly improved bowel regularity and intestinal transit [56,58].

Mineral content is also noteworthy, particularly potassium (K) (650.0–1150.0 mg/100 g), calcium (Ca) (15.42–240.0 mg/100 g), iron (Fe) (0.17–3.00 mg/100 g), copper (Cu) (0.70–0.73 mg/100 g), and manganese (Mn) (1.94–3.27 mg/100 g) [17,59,60]. The relatively high ash content reflects this mineral richness and contributes to the functional positioning of AF as a nutrient-dense ingredient. Based on the mineral concentrations reported, AF may also support specific nutrition claims according to Regulation (EC) 1924/2006. In particular, its potassium, copper, and manganese contents meet the criteria for the claim “high in”, while calcium and iron may qualify as “source of” or “high in”, depending on species and origin [49].

### 3.4. Phytochemicals and Bioactive Compounds in Acorns

AF contains substantial levels of phenolic compounds, including flavanols, gallic acid derivatives, catechin, rutin, ellagic acid, and hydrolysable tannins [23,61,62]. Total polyphenol content has been reported to reach up to 14% in certain species, with tannins accounting for a major fraction [63]. Tannins exhibit antimicrobial, antioxidant, anti-inflammatory, and potential antimutagenic properties [64,65,66].

However, they also contribute to bitterness and may reduce protein digestibility through complex formation [30]. Their dual functional and anti-nutritional roles require careful technological management rather than complete elimination. Notably, acorn phenolics have been shown to reduce starch digestibility and attenuate postprandial glycemic response in model systems [65]. While promising, these findings require further validation in human dietary studies.

### 3.5. Functional Food Potential of Acorns

The functional potential of acorns arises from the ratio of soluble DF to insoluble DF, unsaturated lipids, resistant starch fractions, and polyphenolic antioxidants in a single raw material. This composition supports several functional attributes relevant to modern food formulation:

- Modulation of glycemic response;
- Contribution to antioxidant capacity (AOC);
- Improvement of lipid profile in composite formulations;
- Enhancement of mineral density;
- GF applicability.

Most contemporary research has focused on incorporating AF into bakery and confectionery products, particularly bread, biscuits, cakes, and pasta [23,31,32,67]. These studies consistently demonstrate nutritional enhancement, although technological optimization remains formulation-dependent.

As summarized in Table 1, bakery applications dominate current research, with AF typically incorporated at levels between 10 and 40%, primarily targeting DF enrichment and antioxidant enhancement. Due to its lack of gluten, AF-focused research is mostly dedicated to investigating its influence on GF bakery products, GFB being the most common [23,24,31,32,33,34,35] and followed by biscuits [43,68,69]. Other researched applications of acorn-derived ingredients in functional food systems included acorn oil extract as a functional food present in chocolate spreads [70], roasted and ground acorn kernels [71,72,73] in coffee, as well as ethanol extracts of raw peeled acorns in coffee [74], AF in beer [75] and in milk pudding [76].

**Table 1:** Application of acorn-derived ingredients in functional food systems.

Product Category	Type of Acorn Ingredient	Level of Incorporation	Main Objective	Key Findings	References
Bread	AF	10–45%	Nutritional enrichment, GF formulation, and dough rheology improvement	↑ DF; ↑ AOC; ↓ volume at >30%; ↑ firmness; ↑ viscoelasticity; ↑ cohesiveness; ↑ fatty acids; ↑ minerals	[23,24,31,32,33,34,35]
Wheat bread	AF	5–50%	Technological assessment, nutritional enrichment	↑ dough development; ↓ volume; ↑ water absorption; ↑ DF	[14,77,78,79]
Biscuits	AF	15–45%	Nutritional enrichment, GF formulation, technological assessment	↓ energy value; ↑ AOC; ↑ DF; ↓ volume	[43,68,69]
Iranian toast	AF	10–50%	Nutritional enrichment, technological and rheological assessment	↑ DF; ↑ fat; ↓ degree of softening; ↓ water absorption; ↓ dough extensibility; ↑ dough stability; ↑ development time; ↑ resistance to extension, ↑ AOC	[44]
Pasta	AF	4–25%	Bioavailability, functional (antioxidant) and rheological evaluation	↑ initial polyphenol content; ↑ flavonoid content; ↑ AOC; ↑ free phenolic acids during digestion, improved pasting, mixing and texture	[41,80]
Cake	AF	3–40%	Evaluation of chemical and physical properties	↑ crude DF; ↑ mineral content; ↑ fat; ↑ AOC; ↓ protein, ↓ volume as AF increased; ↑ density and firmness	[42,81,82]
Pita bread	AF	5–15%	Evaluation of rheological and physicochemical properties	↑ DF; ↑ ash; ↑ fat; ↑ protein; ↑ water absorption, ↑ dough stability; ↓ arrival time and dough development time; ↑ firmness	[83]
Muffins	AF	0–70%	Nutritional enrichment, technological evaluation and potential health benefits	↑ AOC; ↓ volume; inflammation improvement	[84,85]
Chocolate spread	Acorn oil extract	14%	Oleogel rheology improvement	↑ physical stability	[70]
Coffee	Roasted/boiled-roasted ground acorn kernels	100%	Nutritional enrichment	↑ mineral content; ↑ AOC	[71,72,73]
Coffee	Ethanol extracts of raw peeled acorns	100%	Potential health benefit evaluation	↑ cholinesterase inhibitors	[74]
Beer	AF	0.2 g/L	Sensory properties assessment	↑ sensory attributes	[75]
Milk pudding	AF	1–7%	Physicochemical properties assessment	↑ AOC; ↑ mineral content; ↑ DF; ↑ hardness; ↑ gumminess	[76]

Abbreviations: AF—Acorn flour; GF—Gluten-free; DF—Dietary fiber; AOC—Antioxidant capacity. Symbols: ↑—increase/higher; ↓—decrease/lower.

Collectively, the evidence indicates that acorn-derived ingredients act as multifunctional food components and simultaneously enhance DF, phenolic and mineral content while exerting measurable technological effects on water binding, dough rheology, structural stability and texture formation. Although high incorporation levels may reduce volume and influence the structure of aerated systems, moderate substitution levels enhance antioxidant capacity, DF content and, in some cases, dough stability and product consistency. Therefore, AF represents a promising ingredient for nutritionally enriched products when applied at optimized inclusion levels.

### 3.6. Acorn Flour: Production and Nutritional Profile

**Figure 1:**
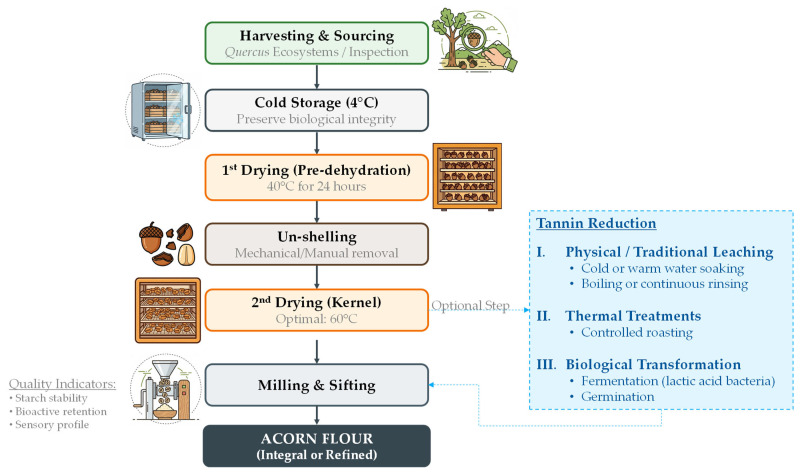
Systematic flowchart of the technological processes in AF production.

AF production involves several critical processing steps that directly influence its nutritional, functional, and sensory characteristics. After harvesting, acorns are typically cleaned, shelled, dried, milled, and, when necessary, subjected to debittering treatments to reduce tannin content (Figure 1) [23,79,86]. Traditional debittering methods include water leaching, boiling, or soaking, while more recent approaches use controlled thermal processing and biotransformation strategies to reduce astringency and modulate phenolic availability rather than completely remove these compounds [3,4].

The production of AF is a specialized, low-input technological process that differs significantly from conventional cereal milling, primarily due to the high initial moisture content, lipid fraction, and tannin levels of the raw feedstock [5,87]. As illustrated in Figure 1, the process follows a structured sequence of operations designed to optimize both starch-related functional properties and overall technological performance of the flour [88].

- Raw Material Sourcing: The process begins with the collection of mature acorns from *Quercus* ecosystems, followed by systematic inspection to remove rotten or infested specimens. Post-harvest handling is critical, as acorns are prone to rapid spoilage due to their high moisture and lipid content. Therefore, raw materials not immediately processed are stored at 4 °C to preserve biological and chemical stability [88,89,90].
- 1st Drying and Un-shelling: Unlike wheat, which undergoes tempering (moisture conditioning), acorns require pre-dehydration at approximately 40 °C for 24 h. This step reduces kernel moisture, limits microbial activity, and facilitates mechanical or manual removal of the pericarp and cupule [89].
- 2nd Drying: After shell removal, acorn kernels undergo a secondary drying phase to further stabilize the material and improve milling efficiency. Reported drying temperatures range from 40 °C to 70 °C; however, 60 °C is often identified as optimal, as it enhances flour consistency, paste stability, and gel-forming capacity, which are critical for baking applications [88].
- Optional Valorization (Debittering and Biotransformation): To enhance palatability, an optional debittering step may be included, involving leaching in water or alkaline solutions to reduce water-soluble tannins responsible for bitterness [4,91]. While these approaches are effective, they may also lead to the loss of soluble nutrients such as minerals and simple sugars. In contrast, thermal treatments primarily induce structural modifications of phenolic compounds (e.g., polymerization or binding to macromolecules), thereby reducing their astringency without complete removal. Emerging strategies such as fermentation (e.g., lactic acid bacteria) and germination promote enzymatic degradation of tannins and modification of the food matrix. These processes enhance functional properties, including water and oil absorption capacity, and reduce tannin bioavailability through interactions with proteins and dietary fiber [90].
- Milling: The final stage involves grinding the processed kernels in laboratory or industrial mills [88,89,90]. Depending on the degree of processing, two main types of flour can be obtained: refined flour, produced exclusively from the kernel, and WG (integral) flour, which utilizes the entire acorn and represents a more sustainable, low-input alternative with higher fiber content [4,92].

The technological processing of acorns plays a decisive role in determining the final nutritional quality of AF, as thermal and mechanical treatments directly affect starch integrity, lipid stability, and the reduction in anti-nutritional compounds. Therefore, optimizing processing parameters is essential to balance sensory acceptability, nutritional value, and functional performance [89,93].

The chemical composition of AF shows pronounced interspecific variability, consistent with studies highlighting the influence of phylogenetic traits and environmental conditions on acorn development and maturation [62]. This compositional diversity enables the targeted selection of oak species according to specific technological requirements in food production systems [21].

As summarized in Table 2, the analyzed species can be broadly categorized into functional groups—lipid-rich, starch-rich, and fiber-rich—which provides a useful framework for standardizing acorn-based ingredients.

Carbohydrates represent the predominant fraction across all analyzed species (Table 2), reaching maximum values in *Q. nigra* (91.92%) and *Q. suber* (up to 85.15%) [21]. Starch is the major carbohydrate component, accounting for up to 50–60% of kernel mass, positioning AF as a relevant energy source [13,21]. Species such as *Q. pubescens*, with carbohydrate content around 78.29%, may exhibit favorable techno-functional properties in bakery applications, where starch plays a key role in crumb formation and dough viscosity [93].

Lipid content is a major differentiating factor among species. Mediterranean evergreen oaks, particularly *Q. ilex* and *Q. rotundifolia*, are classified as lipid-rich, with fat contents reaching up to 13.86% [15]. Their fatty acid profile is dominated by oleic and linoleic acids, contributing to their nutritional value and similarity to olive oil-type lipid profiles [87]. In contrast, species such as *Q. ithaburensis* (0.76%) and *Q. cerris* (up to 5.47%) have substantially lower lipid levels, which may confer improved oxidative stability and greater suitability for products requiring extended shelf life [13,94].

DF content is the most significant functional differentiator among the analyzed species. While several species have moderate DF levels, *Q. petraea* and *Q. ithaburensis* reach exceptionally high values (34.26%, Table 2), markedly exceeding those of conventional wheat flour [13,14]. Such high fiber concentrations enable the formulation of products with “high-fiber” nutritional claims even at moderate substitution levels. However, in gluten-free systems, elevated DF content may adversely affect dough rheology and gas retention [95,96], necessitating process optimization, including hydration adjustment and the incorporation of hydrocolloids such as xanthan gum to maintain acceptable loaf volume [93,97,98].

**Table 2:** Nutritional composition of acorn flours and acorn-derived matrices across *Quercus* species (values expressed as % on a dry weight basis, unless otherwise specified in the original sources).

*Quercus* Species	Sample Type/Matrix	CH (%)	Lipids (%)	Proteins (%)	DF (%)	Ash (%)	Source
*Q. calliprinos*	Whole acorn kernel	77.86	2.31	4.94	13.11	1.78	[13]
*Q. canariensis*	Dehulled kernel flour (air-dried, milled)	77.49–84.87	3.65–4.73	4.59–7.43	2.40–2.60	2.00–2.70	[60]
*Q. cerris*	Whole acorn kernels [94]; dehulled roasted kernel flour (milled, 2–3 mm) [99]	44.10–60.40	4.00–5.47	6.30–10.49	9.11–26.90	2.40–2.52	[94,99]
*Q. faginea*	Acorn pulp—dried and ground for analysis	81.71	7.35	7.03	1.73	2.18	[59]
*Q. ilex*	Dehulled kernel flour (air-dried, milled) [60]; dehulled kernel flour (dried or roasted, milled) [15]	75.22–81.99	7.96–13.86	3.34–5.00	2.67–20.90	1.70–1.96	[15,60]
*Q. ithaburensis*	Whole acorn kernel	58.94	0.76	2.84	34.26	3.21	[13]
*Q. nigra*	Whole acorn (lyophilized, ground)	91.84	2.02	5.26	n.d.	1.25	[100]
*Q. petraea*	Whole acorn kernel [13]; dried whole acorn flour (30 °C, milled)	62.70	4.14	9.27	34.26	2.35	[13,14]
*Q. pubescens*	Dehulled, testa-removed kernel flour (milled, <250 µm)	78.29	5.40	6.50	n.d.	1.91	[93]
*Q. pyrenaica*	Dehulled kernel flour (milled, sieved 1 mm; dehulling variants) [101]; kernel flour and leached kernel flour (milled) [17]	71.11–85.80	4.60–7.19	6.40–8.60	19.70–26.10	2.10–2.82	[17,101]
*Q. robur*	Whole acorn kernels [14,94]; kernel flour and leached kernel flour (milled) [17]; dried acorn tissues (embryo and seed coat), milled (not true flour) [102]	59.17–76.30	5.79–6.08	6.09–10.44	8.61–20.50	1.95–2.84	[14,17,94,102]
*Q. rotundifolia*	Kernel flour and leached kernel flour (milled) [17]; dehulled kernel flour (dried or roasted, milled) [3]	74.56–78.03	11.27–11.75	4.28–4.52	11.40–17.90	1.60–2.00	[3,17]
*Q. rubra*	Whole acorn kernels [14]	56.17	3.09	5.37	n.d.	3.56	[14]
*Q. suber*	Dehulled kernel flour (air-dried, milled) [60]; lyophilized acorn tissues (whole, kernel, pericarp), milled [62]	75.39–85.15	5.20–5.47	5.19–7.63	1.90–2.50	2.13–2.70	[60,62]

CH—carbohydrates; DF—Dietary fiber.

Although protein content in AF is generally low (approximately 2–8% across most species), certain species, such as *Q. cerris*, exhibit higher values (up to 10.49%, Table 2), approaching those found in some cereal flours [94]. To our knowledge, there are no studies that have investigated the amino acid profile of AF directly. Özcan (2006) identified all essential amino acids in whole acorns used for flour production, leucine and lysine (245 mg/100 g d.m. and 208 mg/100 g d.m.) appearing in the largest amounts [103]. However, the content of essential amino acids like leucine and lysine is two to three times lower than that in whole grain wheat flour [104]. Importantly, AF is naturally gluten-free, making it a suitable raw material for products intended for individuals with celiac disease or gluten intolerance [31]. In addition to macronutrients, AF contains appreciable levels of minerals, as reflected in ash content, which reaches up to 3.56% in *Q. rubra* (Table 2) [14]. This indicates a substantial presence of essential minerals such as potassium, iron, and calcium, supporting the potential of AF to contribute to dietary mineral intake [19]. The compositional data presented in Table 2 support the classification of acorn species into three functional categories: (i) lipid-rich (*Q. rotundifolia*, *Q. ilex*), (ii) starch-rich (*Q. robur*, *Q. pyrenaica*, *Q. pubescens*), and (iii) fiber-rich (*Q. ithaburensis*, *Q. petraea*, *Q. cerris*). This classification provides a practical basis for the selection and standardization of raw materials in the industrial production of acorn-enriched bakery products.

AF has a unique macronutrient profile compared to cereal flours. Table 3 shows that **moisture** in AF is generally lower (about 3.8–11.8% in lipid- and starch-rich AF) than in wheat or rye (approximately 11.7–14.7%) [17,18,60]. Total carbohydrates predominate in AF, ranging from roughly 44–92% across species (overlapping with values for rice and corn), confirming AF as a substantial energy source [16,105]. Starch is the main carbohydrate in AF (constituting the majority of the carbohydrate fraction), although high processing temperatures can partially break down starch into sugars [13,21,89]. Protein in AF (approximately 2.8–10.5%) is generally lower than in wheat (about 9.9–14.6%) but comparable to rice and corn (Table 3) [20,106]. Importantly, AF proteins are naturally gluten-free, making AF suitable for gluten-free product formulations, though their limited quantity and lack of gluten functionality often require recipe adjustments, such as blending with other flours or using binders [20,93]. Lipid content is one of the distinguishing features of AF. Lipid-rich species (*Q. ilex*, *Q. rotundifolia*) contain 7.03–13.86% fat, much higher than the approximately 1.5–3.6% found in wheat [15,16]. Their fatty acid profile is rich in monounsaturated fats (oleic acid up to 59.9–67.0%) and linoleic acid, similar to olive oil. This enhances nutritional value but also increases susceptibility to oxidation [17,61]. In contrast, low-fat species (e.g., *Q. cerris* with 0.76–5.47% fat) offer greater oxidative stability and are better suited for long-shelf-life products. Technological implication: High-fat AF may require antioxidant protection (such as tocopherols or inert packaging), whereas low-fat AF can be handled like traditional flours. Fiber is the most notable functional difference. Fiber-rich AF (e.g., *Q. ithaburensis*, *Q. petraea*, *Q. cerris*) contains 26.90–34.26% dietary fiber, much higher than refined flours and even whole grain wheat (11.42–14.10%) [13,99]. Starch-rich and lipid-rich AF types have moderate fiber (about 6–22%), similar to whole grain rye (16.7–19.0%) [20]. The high fiber content improves nutritional profile but can impair dough properties by absorbing water and weakening gas retention. Gluten-free AF breads with very high fiber typically require careful hydration and additives such as xanthan gum or hydroxypro-pylmethylcellulose (HPMC) to maintain loaf volume [34,93,107]. AF is also richer in certain micronutrients than wheat. For example, vitamin E in AF is about 15–20 mg/100 g, whereas wheat and rye have about 0.6–0.8 mg/100 g [17,18]. AF also contains substantial minerals: potassium and calcium are often higher than in cereals (e.g., potassium up to about 1000 mg/100 g vs. about 360 mg in wheat) [19,20]. These enhanced micronutrient levels further highlight AF’s potential as a functional ingredient [61,63,108].

**Table 3:** Chemical composition of functional AF groups in comparison to conventional flours.

Compound (Unit)	Lipid-Rich AF	Starch-Rich AF	Fiber-Rich AF	WG Wheat Flour	WG Rye Flour	Rice Flour	Corn Flour
**Water (%)**	4.94–11.76 [15,17,60]	3.81–11.77 [17,60,93]	5.20–7.36 [13,94]	11.70–14.70 [18,109]	10.00–14.45 [18,110,111]	8.35–12.80 [20,106]	10.10–14.00 [16,20,105]
**Carbohydrates (%)**	70.33–84.09 [3,15,17]	71.11–84.87 [17,60,93]	44.10–62.70 [14,94,99]	63.90–82.40 [109,112]	82.70 [109]	79.17–82.13 [106]	74.30–92.01 [16,105]
**Sugars (%)**	1.70–34.11 [17,60,108]	1.20–18.54 [17,60]	5.21 [13]	2.10 [112]	1.80 [111]	n.d.	0.60 [16]
**Proteins (%)**	3.34–5.19 [3,15,60]	4.59–10.44 [17,60,93,94]	2.84–10.49 [13,94,99]	9.89–14.60 [20,109,110]	7.31–13.80 [109,110,111]	6.89–9.43 [20,106]	5.50–9.40 [16,20,105]
**Lipids (%)**	7.03–13.86 [15,59,60]	3.65–7.19 [17,60,93,94]	0.76–5.47 [13,14,94]	1.51–3.63 [20,109,112]	1.31–2.10 [109,110,111]	0.16–2.45 [20,106]	2.48–12.23 [16,20,105]
**Main fatty acids (%)**							
**Palmitic acid**	12.52–15.10 [15,60,61]	13.12–14.76 [17,60]	n.d.	16.30–19.74 [18,20]	13.40–19.41 [18,113]	22.43 [20]	9.20–12.62 [20,114]
**Oleic acid**	59.85–67.00 [3,15,61]	20.82–56.84 [17,60]	n.d.	12.73–14.00 [18,20]	16.20–17.34 [18,113]	40.01 [20]	19.50–30.50 [20,114]
**Linoleic acid**	14.29–16.12 [3,15,61]	6.62–24.07 [17,60]	n.d.	60.79–61.60 [18,20]	54.58–55.80 [18,113]	29.38 [20]	53.00–65.30 [20,114]
**DF (%)**	10.89–22.81 [3,15,17]	6.40–31.16 [17,93,101]	26.90–34.26 [13,99]	11.42–14.10 [18,20,110]	16.71–19.00 [18,111,115],	0.21–0.87 [20,106]	2.62–7.30 [16,20]
**Ash (%)**	1.60–2.73 [3,21,60]	1.91–2.99 [17,93,94]	0.95–3.21 [13,14,33]	1.32–1.74 [20,109,110]	1.27–2.00 [109,110,111]	0.25–1.25 [20,106]	0.02–1.20 [16,20,105]
**Tannins (%)**	3.05–3.81 [61,108,116]	0.57–3.39 [14,17]	1.05–3.73 [13,14]	0.57 [117]	0.146 [117]	n.d.	0.427 [117]
**Vitamins (mg/100 g)**							
**E**	17.06 [17]	15.96–20.71 [17]	15.45 [99]	0.63 [18]	0.77 [18]	20.90–29.90 [118]	94.10 [117]
**B1**	0.02 [119]	n.d.	n.d.	0.27–0.86 [18,112,120]	0.343–0.42 [18,115]	0.01–0.13 [119]	0.39 [16]
**B2**	0.13 [119]	n.d.	n.d.	0.09–0.24 [18,112,120]	0.102–0.128 [18,115]	0.10 [119]	0.20 [16]
**B6**	0.03 [119]	n.d.	n.d.	0.01 [16]	n.d.	0.15 [119]	0.62 [16]
**Minerals (mg/100 g)**							
**Calcium (Ca)**	15.42–240 [17,59,60]	28.24–164 [17,60,102]	30.77–380 [13,14,33]	30.77–39.00 [18,20,121]	36.90–37.00 [18,121]	5.07 [20]	3.32–7.00 [16,20]
**Iron (Fe)**	0.17–3.00 [3,17,60]	0.20–19.7 [17,19,102]	0.76–2.54 [14,33]	2.69–3.40 [18,20,121]	2.30–2.36 [18,121]	0.60 [20]	0.91–2.70 [16,20]
**Potassium (K)**	650.0–1150 [3,60]	713.1–1095 [17,19,102]	148.8–1030 [13,14,33]	360.0–399.77 [18,20,121]	440.0–480.0 [18,121]	97.37 [20]	148.70–287.0 [16,20]
**Zinc (Zn)**	0.37–1.30 [3,17,60]	0.13–10.40 [17,19,102]	0.62 [14]	1.75–3.90 [18,20,121]	2.35–3.00 [18,121]	1.78 [20]	0.66–2.20 [16,20]

Values are expressed as means or ranges based on the cited literature. Lipid-Rich AF: *Q. ilex*, *Q. rotundifolia*; Starch-Rich AF: *Q. robur*, *Q. pyrenaica*, *Q. canariensis*, *Q. pubescens*; Fiber-Rich AF: *Q. ithaburensis*, *Q. petraea*, *Q. cerris*); AF—Acorn flour; GF—Gluten-free; WG—Whole grain; DF—Dietary fiber; n.d.—not determined/no data available.

Although not fully detailed in Table 3, AF is known to contain tannins and phenolics. Tannins (up to about 3.8% in lipid-rich AF) may impart bitterness and reduce protein digestibility, but they also contribute antioxidant and anti-inflammatory effects [63,108]. Notably, tannins slow starch hydrolysis, potentially lowering postprandial glycemic response [61,108]. These bioactive compounds enhance the functional and nutritional value of AF but their levels must be carefully managed by processing techniques (e.g., soaking or fermentation) to improve palatability.

AF (depending on species) is high in energy (from starch), lipid-rich (in some types), and often fiber-rich. These data-driven findings underscore that species selection is critical: lipid-rich oaks (e.g., *Q. ilex*) yield flours similar to high-fat ingredients, starch-rich oaks (e.g., *Q. robur*, *Q. pyrenaica*) provide starch functionality, and fiber-rich oaks (e.g., *Q. ithaburensis*, *Q. petraea*) produce highly fibrous flour. Each group has distinct technological implications (oxidation stability, dough handling, nutrient fortification) that must be addressed during product development.

## 4. Innovative Food Product: Acorn Bread

AF, despite its historical use, has recently gained renewed attention in scientific research as an innovative ingredient for breadmaking. Traditional acorn flour bread (AFB) preparations date back centuries, originating in Sardinia, where acorns were processed using prolonged drying and leaching techniques to reduce tannins and enhance mineral content [122].

Similar historical practices were documented among Native American populations in California, such as the Shasta Indians, who produced “xaro”—a sweet, black AFB obtained after leaching and baking acorn meal mixed with iron-rich soil [123,124,125,126]. These traditional methods underscore the long-standing dietary and cultural relevance of AFB.

In contemporary contexts, AF is primarily explored for GFB production due to its lack of gluten and its ability to improve nutritional quality. Gluten, a protein complex responsible for dough elasticity and gas retention, is associated with various gluten-related disorders, including celiac disease, non-celiac gluten sensitivity, wheat allergy, gluten-induced enteropathy, gluten ataxia, and dermatitis herpetiformis [127,128,129]. The increasing prevalence of these conditions, along with rising global gluten consumption, drives demand for high-quality GF bakery products [130].

GFBs generally suffer from technological and sensory limitations, including low specific volume, crumb dryness, rapid staling, and reduced nutritional value, largely due to weak protein networks and limited gas retention during fermentation [36,107]. Introducing non-wheat flours—such as rice, maize, sorghum, buckwheat, amaranth, quinoa, or AF—can enhance protein, essential amino acids, vitamins, minerals, and DF, improving both nutritional and functional properties [55,56,131].

AF offers additional advantages:

- It does not require novel food authorization in many jurisdictions.
- It can diversify plant-based ingredient portfolios for bakery products.
- It contributes bioactive compounds, including polyphenols, tocopherols, and carotenoids [31].

Recent advances in AFB research are summarized in Table 4, highlighting species, substitution levels, formulations, procedural variations, and observed impacts on nutritional, technological, and sensory properties.

The data presented in Table 4 highlight the dual role of AF as both a nutritional fortifier and a rheological modifier in bread production. Across various studies, a clear distinction emerges between its application in GF systems and wheat-based matrices.

1. Impact on Nutritional and Bioactive Profiles: AF significantly enhances the functional value of bread, particularly by introducing healthy lipid fractions (MUFAs and PUFAs) and essential minerals. Research by Beltrão Martins et al. (2020a, 2022b) [3,23] shows that *Q. rotundifolia* is especially effective in increasing the antioxidant potential of GFB. Additionally, the integration of sourdough fermentation is a crucial strategy for mitigating the high glycemic index often associated with GF products by reducing starch hydrolysis [23,24].
2. Rheological and Textural Challenges: Technologically, incorporating AF presents a trade-off between nutrition and structure. In wheat-based systems, AF tends to weaken the gluten network, resulting in lower loaf volumes and increased crumb firmness [78,79]. In contrast, in GF formulations, the high DF and starch content of acorns can strengthen the dough matrix, as shown by the increased storage modulus and stability observed by Korus et al. (2015) and Szabłowska and Tańska (2025a) [31,77].
3. Sensory Acceptance and Substitution Thresholds: The literature suggests a sensory threshold for acorn substitution. Lower levels (up to 23%) generally improve color through enhanced browning and provide desirable nutty notes, while higher concentrations (>30%) often introduce bitterness and excessive density. This is particularly evident in *Q. ithaburensis* and generic species, where high substitution levels negatively affected physical quality despite nutritional gains [33].

The strategic use of specific species such as *Q. rotundifolia*, combined with processing techniques like sourdough fermentation or inulin addition, enables the development of acorn-enriched breads that meet both the nutritional requirements and sensory expectations of modern consumers.

Overall, despite variability in species and formulations, a consistent trend emerges in which moderate acorn flour substitution (approximately 20–35%) provides the most balanced improvement in nutritional and technological performance. However, the comparability of studies is limited by differences in processing conditions, flour pre-treatment, and analytical methods, which complicates the establishment of standardized formulation guidelines.

### 4.1. Breadmaking Process with Acorn Flour

The inclusion of AF in bread formulations can involve:

- Sifting—incorporates air and removes impurities, improving hydration and dough consistency [132].
- Mixing—speed, duration, and geometry influence aeration, fermentation efficiency, and dough rheology. Longer mixing is typically applied in GF AFB to enhance aeration and gas retention [23,24,31,33,34,133].
- Flour blends—AF is often combined with rice flour, buckwheat, or other starches to optimize dough structure and sensory attributes [23,24,33,34,35]. Rice flour provides a neutral taste, white color, hypoallergenic carbohydrates, and ease of digestion, while buckwheat contributes protein and favorable amino acid profiles [134,135].
- Hydrocolloids—HPMC, guar gum, xanthan gum and pectin improve structure and mimic gluten functionality, enhancing moisture retention, crumb texture, loaf volume, and elasticity [23,31,34,98,136].
- Fermentation—typically yeast-based (*Saccharomyces cerevisiae*) or sourdough; parameters such as temperature (30–37 °C) and relative humidity (~85%) are critical for optimal gas production. Mixed fermentation strategies combining sourdough and prebiotics (e.g., inulin) have shown enhanced gas retention and crumb structure [24,34,35].
- Baking—180–240 °C; key physicochemical transformations occur, including water evaporation, starch gelatinization, protein denaturation, Maillard reactions, and crust formation [23,24,78,137].

Moderate AF inclusion generally supports dough structure and volume, while excessive substitution may reduce loaf volume due to increased density and impaired gas retention [23,31,35].

### 4.2. Acorn Bread Properties

#### 4.2.1. Nutritional Profile

Acorn-enriched breads show increased DF, mineral content, UFAs, and bioactive compounds, especially when AF is incorporated at 20–35% in GF formulations [23,24,31,33,34].

Key findings include:

- Lipids: Oleic (~60%) and linoleic (~16%) acids increase total UFAs [23,34].
- Minerals: Potassium, calcium, manganese, iron, magnesium, and phosphorus contents increase proportionally with acorn substitution [23,33,138].
- DF: Total and insoluble DF content rises substantially, improving nutritional and functional properties [24,56].

#### 4.2.2. Sensory Attributes

AF imparts a characteristic nutty flavor and darker crumb color, generally perceived positively in GF and WG contexts [23,35]. Optimal substitution levels (20–40%) maximize acceptability; higher levels may cause bitterness due to tannins or affect texture [31,35].

In wheat-based breads, limited acorn incorporation (≤18%) maintains sensory quality, with some improvement when blended with barley flour [78].

#### 4.2.3. Rheological and Technological Characteristics

AF alters dough rheology depending on species, flour blend, and substitution level:

- Moderate inclusion (20–30%) can increase elasticity and gel-like structure in GF doughs [24,35].
- Excessive levels (>35%) may reduce elasticity and volume due to weakened structure [24,31].
- Crumb texture: Hardness increases with DF content, while cohesiveness and springiness depend on formulation [23,33,34,35].
- Staling: Acorn-enriched breads, especially at moderate inclusion, may stale more slowly due to water retention and DF-starch interactions [31,33].

Crumb color changes are consistent with Maillard reactions and phenolic content, resulting in darker, more reddish breads preferred by consumers of GF and health-oriented products [35,139,140]. Taken together, the findings indicate that acorn flour shows a multifactorial influence on bread systems, where improvements in nutritional composition are often accompanied by the opposite effects in technological performance and sensory attributes. A relatively consistent pattern across studies suggests that moderate substitution levels (approximately 20–35%) represent a critical range for balancing these effects. However, the diversity of acorn species, flour pre-treatments (e.g., leaching, roasting), and formulation strategies introduces significant variability and limits direct comparability between studies. In addition, most available data are derived from controlled experimental conditions, highlighting the need for further research under standardized protocols and industrialized processing environments.

### 4.3. Functional and Potential Health Benefits

AF provides bioactive compounds, including polyphenols, tocopherols, carotenoids, triterpenes, and sterols, which may have antioxidant, anti-inflammatory, and enzyme-modulatory effects [23,141,142,143]:

- Polyphenols: Gallic acid, ellagic acid, quercetin, and azelaic acid inhibit α-amylase, α-glucosidase, and dipeptidyl peptidase IV in vitro, potentially influencing glucose metabolism [144,145,146].
- Antioxidants: γ-tocopherol, β-carotene, and lycopene trap reactive oxygen and nitrogen species, supporting cardiovascular health [12,62,147,148,149].
- Triterpenes and terpenoids: Show anti-proliferative, anti-fibrotic, and anti-inflammatory effects [149,150,151].

These effects are mainly based on in vitro or model system studies; further research is needed to confirm functional benefits in human diets.

**Figure 2:**
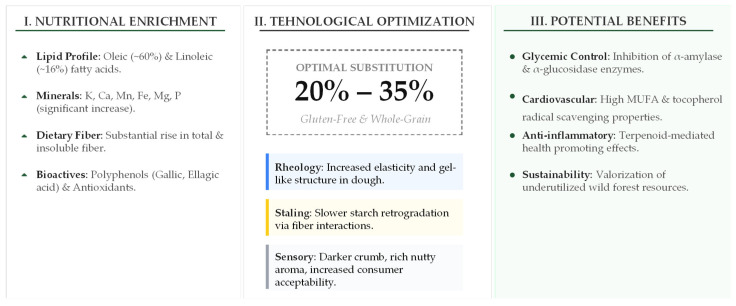
Integrative overview of the nutritional, technological, and functional development of acorn-enriched bread.

The multifaceted impact of AF on bread properties is complex, involving a delicate balance between chemical enrichment and structural integrity. A comprehensive synthesis of these interactions—from the raw nutritional input to the resulting technological performance and potential physiological outcomes—is illustrated in Figure 2.

The schematic illustrates the progression from raw material input (Panel I: Nutritional Enrichment) and dough matrix optimization (Panel II: Technological Properties) to the expected health-related outcomes (Panel III: Potential Benefits). The central axis indicates the optimal substitution level (20–35%) needed to maximize bioactive delivery while maintaining the rheological stability and sensory profile of the final bakery product.

As shown in Figure 2, the optimal substitution range (20–35%) serves as a critical threshold. Within this range, the bread achieves significant enrichment in MUFAs (oleic acid) and essential minerals (K, Ca, Fe), while also benefiting from improved shelf life (reduced staling) and enhanced sensory appeal through darker crumb pigmentation and a distinctive nutty aroma [23,24,31,33,35]. Additionally, the presence of specific polyphenols (such as gallic and ellagic acids) correlates with observed modulatory effects on carbohydrate-digesting enzymes [144,145,146], positioning acorn-enriched bread as a viable functional food candidate within the circular bioeconomy framework.

While these findings highlight promising functional properties, the current evidence is mostly based on in vitro and model systems and their translation into clinical outcomes remains uncertain. This represents a key gap that should be addressed through human intervention studies.

### 4.4. Consumer and Sustainability Perspective

Taken together, the available evidence positions AF as a promising multifunctional ingredient; however, its successful implementation in bakery systems depends on carefully balancing nutritional enhancement with technological performance and sensory acceptance.

AFB offers nutritional enrichment, sensory appeal, and sustainable sourcing. Consumer interest is likely among GF populations, elderly individuals, and health-conscious or environmentally aware groups. Acorns remain underutilized wild resources. Their use in breadmaking supports diversification of staple foods, reduces reliance on conventional cereals, and aligns with sustainable food system goals [46,47].

## 5. Sustainability and Organic Production Perspective of Acorn Bread in the EU

### 5.1. Introduction and the EU Strategic Framework

The integration of alternative raw materials, such as AF, into modern food systems aligns with the objectives of the European Green Deal [152] and the Farm to Fork Strategy [153]. These frameworks promote a transition toward Sustainable Food Systems (SFS) by reducing dependence on intensive monoculture and encouraging the use of neglected and underutilized species (NUS).

AFB may be considered a “low-input” product model derived from resilient, naturally occurring biomass with minimal human intervention [47]. Additionally, the proposed Legislative Framework for Sustainable Food Systems (FSFS) seeks to establish horizontal sustainability requirements, with acorn-based products supporting agro-biodiversity and requiring less energy than conventional cereals.

### 5.2. Ecological Characteristics and Environmental Impact

Oak forests (*Quercus* spp.) are structurally complex, biodiverse ecosystems widely distributed across Europe [1,4]. Unlike annual cereal systems, oak stands function as perennial agroforestry systems that do not require synthetic fertilizers, irrigation, or pesticides [154]. The environmental advantages of these systems are quantitatively supported by comparative analyses of their ecological footprints.

**Figure 3:**
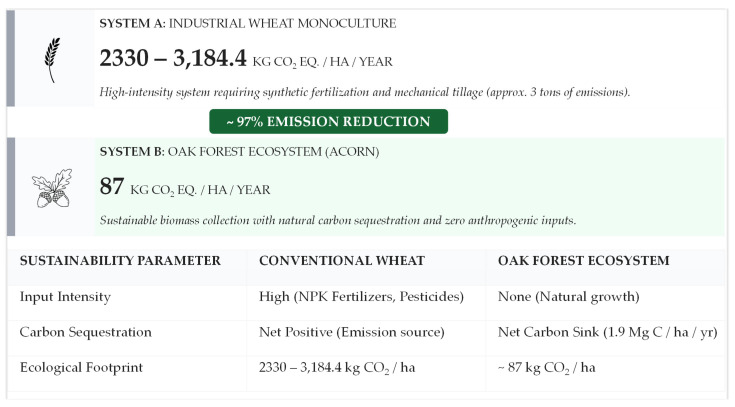
Comparison of environmental footprints: Industrial wheat systems vs. oak agroforestry.

Figure 3 presents key sustainability metrics comparing conventional wheat production with oak forest ecosystems, highlighting a substantial reduction in environmental impact under the reported conditions [38].

As shown in Figure 3, the conventional wheat system generates nearly 3000 kg CO_2_ ha^−1^ yr^−1^ (ranging from approximately 2330 kg CO_2_-eq ha^−1^ yr^−1^ [155] to 3184.4 kg CO_2_-eq ha^−1^ yr^−1^ [156], depending on the intensity of fertilization and irrigation. In contrast, oak ecosystems, operating as low-input perennial systems, are characterized by a minimal carbon footprint (estimated at 87 kg CO_2_-eq ha^−1^ yr^−1^) due to the absence of mechanical tillage and nitrogen-based fertilizers, which are the primary drivers of emissions in cereal monocultures [154,156]. These values should be interpreted as indicative comparative estimates based on available literature rather than universally transferable LCA benchmarks.

Additionally, cereal crops contribute to a rapid, net-positive carbon cycle, whereas perennial oak systems enable long-term carbon sequestration, serving as important carbon sinks that support EU climate neutrality targets [38,157]. Oak forests exhibit a carbon sequestration potential of approximately 1.9 Mg C ha^−1^ yr^−1^ [157] with mature oak-dominated woodlands capable of capturing roughly 18 tons of CO_2_ per hectare annually [158].

However, these comparisons should be interpreted with caution. Variability in site-specific conditions, forest management practices and post-harvest processing requirements may influence the overall environmental performance of acorn-based systems [154,159].

### 5.3. Regulatory Compliance and Organic Certification

The primary legal framework is Regulation (EU) 2018/848 [37], which promotes biodiversity and the protection of natural resources. Acorns harvested from certified forests are classified as “wild plant products.” In the Republic of Croatia, this is further supported by the Agriculture Act (*Zakon o poljoprivredi*, NN 118/18, 152/22).

While wild collection is explicitly permitted if habitat stability is maintained, challenges persist regarding the standardization of harvesting, traceability of wild-collected material, and monitoring of environmental contaminants. Currently, the absence of harmonized EU-level technical guidelines for acorn processing creates a “regulatory grey zone” that requires future clarification. This lack of harmonization may pose challenges for large-scale commercialization and highlights the need for clearer regulatory frameworks to support the development of acorn-based food products.

### 5.4. Circularity, Nutrition, and Public Health

Valorizing acorns aligns with circular economy principles by utilizing underused forest biomass and reducing dependence on imported GF raw materials [11]. From a health perspective, AF offers:

- High DF: 10–18% [15].
- Healthy fats: High oleic acid content (~60%) [50].
- Bioactive compounds: Phenolic compounds with high antioxidant potential [23,62].
- Minerals: Essential levels of calcium, iron, copper and manganese.

These properties support reduced glycemic responses and cardiometabolic benefits [64,160]. However, due to tannin variability (7–11%) and lower protein content compared to legumes, AF is best used as a functional enrichment strategy (15–35% substitution) rather than a total replacement for cereal flours [31,63].

### 5.5. Technological Feasibility in Organic Systems

Organic baking restricts synthetic additives, making the inherent properties of AF essential. Research shows that AF increases water absorption and dough stability [77] and alters rheological behavior [24]. In GF systems, DF–starch interactions strengthen structure, though excessive levels can impair gas retention [31]. Optimized strategies compatible with organic standards include:

- Sourdough fermentation to improve sensory and structural profiles [34].
- Controlled drying temperatures to preserve bioactive integrity [88].

Importantly, these technological considerations are closely linked to both environmental and regulatory aspects. Simultaneously, the processing intensity, ingredient selection and formulation strategies directly influence the sustainability profile of the final product and its compliance with organic production standards.

### 5.6. Socio-Economic Benefits and Short Food Supply Chains (SFSC)

AFB production supports SFSC, minimizing food miles and allowing primary producers to retain more market value [161]. Using local non-wood forest products (NWFPs) stimulates rural employment and regional food sovereignty, meeting consumer demands for transparency and traceability.

### 5.7. Critical Research Gaps and Future Directions

To advance from experimental formulations to system-level food innovation, the following gaps must be addressed:

- Empirical LCA Data: Comparative Life Cycle Assessments (cradle-to-gate) are needed to quantify the energy demands of debittering and milling compared to conventional flour.
- Standardization: Establishing species-specific quality benchmarks for tannin content and microbiological safety of forest-harvested nuts is essential for industrial scalability.
- Safety Thresholds: Validating protocols to ensure the absence of mycotoxins and heavy metals in wild-harvested batches.

The organic production of AFB represents a potential synergy between traditional knowledge on ecology and modern EU frameworks regarding sustainability. The relatively low ecological footprint of oak species compared to conventional cereal systems, combined with their resilience, together support their consideration with sustainable food strategies. However, further work is required to address technological standardization, regulatory clarity and safety assurance before large-scale implementation. By diversifying flour sources and exploring low-input perennial systems, acorn-based products may contribute to enhancing food system resilience within the EU.

## 6. Conclusions

The current literature confirms that AF is an environmentally promising and nutritionally superior ingredient for bakery applications, particularly due to its rich mineral profile (K, Ca, Fe, Mg), high MUFA content (especially oleic acid), and significant antioxidant capacity. Its integration into breadmaking aligns with EU organic production standards, supporting biodiversity and sustainable land management within a circular bioeconomy framework.

However, several challenges remain, primarily concerning the compositional variability of AF across different *Quercus* species and geographical origins, which complicates the establishment of standardized nutritional benchmarks. Furthermore, significant research gaps persist regarding the long-term stability of acorn-enriched products, the bioavailability of their minerals in the presence of tannins, and the lack of clinical studies confirming their functional health benefits in humans.

To transition from experimental formulations to industrial innovation, future efforts should focus on standardized species profiling and the development of pilot-scale production strategies. Strategically targeting the 20–35% substitution range allows for the creation of functional, organic, and sustainable bakery products that meet modern consumer demands, provided that safety aspects such as potential mycotoxin contamination and heavy metal accumulation in raw materials are adequately monitored and controlled. Ultimately, AF bread shows strong potential to transform a niche traditional product into a scientifically validated and commercially viable alternative that enhances the resilience of the EU food system.

## Figures and Tables

**Table 4:** Impact of AF substitution on dough rheology and bread quality parameters across different matrices.

Acorn Species	Substitution Level	Matrix and Process Conditions	Main Effects on Dough and Bread Quality	Reference
*Q. rotundifolia*	23%, 35%	GF (buckwheat/rice); Baking: 180 °C, 50 min	Enhanced bioactive profile (lipids, minerals); 23% addition optimized sensory acceptance.	[23]
*Q. rotundifolia*	23%, 35%	GF (rice); Sourdough fermentation	The synergistic effect of acorn and sourdough reduced starch hydrolysis and improved fatty acid profile.	[34]
*Q. rubra*, *Q. robur*, *Q. petraea*	5%, 10%, 15%	Wheat bread (type 650 flour); double fermentation, standard baking	Increased crumb hardness and density at higher levels; darker crust/crumb (especially red oak); reduced sensory acceptance due to bitterness (tannins); acceptable quality at 5% substitution	[79]
*Q. ilex* and *Q. rotundifolia*	23%, 35%	GF (buckwheat/rice); Rheological characterization	Increased dough viscoelasticity and firmness; significantly modified pasting properties.	[24,33]
*Q. ithaburensis*	15%, 30%, 45%	GF (rice/corn); Fermented vs. raw flour	Fermentation enhanced nutritional density but negatively impacted loaf volume and texture.	[33]
*Quercus sp*. (n.d.)	20%, 40%, 60%	GF (corn/potato starch); Double fermentation	Strengthened starch network; increased storage modulus; improved overall sensory scores.	[31]
*Quercus* sp.	10%, 30%, 50%	GF (rice); Inulin addition; 4 h incubation	Improved crust browning; significant changes in crumb technological and antioxidant parameters.	[35]
*Quercus* sp.	5%, 10%	Wheat and Wheat-Barley composites	Increased water absorption; weakened gluten network; bitter/acidic notes at >10% substitution.	[78]
*Q. robur*/ *Q. petraea*	0–100%	Wheat-acorn blends (up to 50:50)	Enhanced dough stability and development time; reduced specific volume and flowability.	[77]

Substitution levels are expressed as a percentage (%) of flour replacement. Abbreviations: AF—Acorn flour; GF—Gluten-free; n.d.—not defined (species not specified in the original study). Symbols: >: greater than.

## Data Availability

The original contributions presented in this study are included in the article. Further inquiries can be directed to the corresponding author.

## Abbreviations

The following abbreviations are used in this manuscript:

AF	Acorn flour
AFB	Acorn flour bread
AOC	Antioxidant capacity
DF	Dietary fiber
*DM*	Dry matter
*dw*	Dry weight
EU	European Union
FAME	Fatty acid methyl-esters
FSFS	Framework for Sustainable food systems
GF	Gluten-free
GFB	Gluten-free bread
MUFAs	Monounsaturated fatty acids
NUS	Neglected and underutilized species
PRISMA	Preferred Reporting Items for Systematic Reviews and Meta-Analyses
SFS	Sustainable food systems
UFAs	Unsaturated fatty acids
WG	Whole grain
